# The auditory function in migraine model rats induced by postauricular nitroglycerin injection

**DOI:** 10.3389/fneur.2023.1259982

**Published:** 2023-10-25

**Authors:** Rongxiang Qi, Jilei Zhang, Tongxiang Diao, Lisheng Yu

**Affiliations:** Department of Otolaryngology, Head and Neck Surgery, People's Hospital, Peking University, Beijing, China

**Keywords:** postauricular injection, intraperitoneal injection, auditory function, migraine model rat, cochlear migraine

## Abstract

**Objective:**

The mechanism by which migraines produce inner ear-related symptoms is not well understood. Previous studies have found that the latency of auditory brainstem response (ABR) in animal models of migraine has changed, but the threshold has not changed significantly. Therefore, it is necessary to establish a better animal model with both migraine and hearing loss to explore the relationship between migraine and auditory function deeply.

**Methods:**

In this study, the rat model of migraine was induced by postauricular injection of nitroglycerin (NTG), and the effect on the auditory function of the inner ear was explored by comparing with intraperitoneal injection of nitroglycerin. The rats were given the drug repeatedly on alternate days, a total of 5 dosing, with the body weight monitored during the drug administration. The tactile threshold of the rats' forepaw was measured using von-Frey filaments and auditory function was assessed by ABR.

**Results:**

The results showed that the baseline tactile threshold of rats gradually decreased during the modeling process, and hyperalgesia appeared. Postauricular injection of NTG did not affect the weight gain of rats, while intraperitoneal injection of NTG showed slow or even negative weight gain. The ABR threshold of Click, 4 and 8 kHz of postauricular NTG injection rats increased, the latency was prolonged, and the ABR threshold in the right ear was higher than that in the left ear.

**Conclusions:**

We demonstrated that postauricular injection of nitroglycerin may be safer and more effective than intraperitoneal injection of nitroglycerin in the process of creating rat migraine model without affecting the weight gain. Postauricular injection of nitroglycerin has more damage to the auditory function of rats. Therefore, the migraine model rat induced by postauricular injection of nitroglycerin may be a new model of cochlear migraine.

## 1. Introduction

Migraine is a common neurological disease, which is characterized by paroxysmal, mostly unilateral, moderate to severe throb headache, often accompanied by photophobia, phonophobia, nausea and vomiting (1). According to the global burden of diseases 2016 study, migraine is the second most common neurological disability disease (2). Migraine patients can be accompanied by inner ear related symptoms such as vertigo, hearing loss, tinnitus, hyperacusis, ear fullness, etc. The incidence of hearing loss in migraine patients ranges from 3.3 to 14% (3, 4); however, there are few studies using migraine animal models to explore the relationship between migraine and the inner ear function, especially those related to auditory function. Arakaki et al. (5) reported that the latency of IV, V, and VI waves of the ABR of the 8-khz stimulus sound was significantly prolonged 2 h after nitroglycerin administration in the rat model of migraine. Therefore, to understand the association between migraine and inner ear hearing impairment deeply, it is necessary to establish a better animal models with both migraine-related manifestations and hearing loss.

Postauricular injection is a new method of drug administration. At present, postauricular injection of glucocorticoids is mainly used to treat inner ear diseases such as sudden deafness, tinnitus, and ear fullness. Previous studies have shown that postauricular injection has the advantages of being less invasive, convenient, and having high local cochlear drug concentrations (6, 7). Qiu et al. (8) used the anti-tumor drug cisplatin to explore the drug distribution characteristics after postauricular injection in animal experiments, the results showed that the systemic adverse reactions induced by cisplatin in the postauricular injection group and the intraperitoneal injection group were similar, and the postauricular injection of cisplatin could cause obvious damage to bilateral cochlear hair cells, and the damage of hair cells in the ipsilateral cochlea was significantly greater than that in the contralateral cochlea, postauricular injection of cisplatin also caused more damage to the hair cells of the ipsilateral cochlea than intraperitoneal injection, it is suggested that postauricular administration can achieve higher local drug concentration than systemic administration. In general, postauricular injection has the characteristics of high drug concentration in the inner ear of the administration side and drug distribution to the opposite inner ear and the whole body.

The nitroglycerin-induced migraine model is a classic experimental migraine model. The methods of nitroglycerin injection include intraperitoneal injection, intravenous injection and subcutaneous injection, and intraperitoneal injection is the most commonly used. So far, no relevant literature and reports on migraine animal models by postauricular injection of nitroglycerin have been retrieved. According to the distribution characteristics of drugs after postauricular injection, the migraine model rat was established by postauricular injection of nitroglycerin, so as to better study the correlation between migraine and the inner ear.

## 2. Material and methods

### 2.1. Animal subjects

Healthy male Wistar rats (7–8 weeks old, 200–250 g) were purchased from SPF Biotech Ltd (Beijing, China). The animals were housed under standard laboratory conditions: 20 ± 4°C ambient temperature with a relative humidity of 60 ± 5% and a 12-h light/dark cycle. All the animals had unlimited access to food and water. This study was approved by the Animal Ethics Committee of Peking University People's Hospital (Approval Number: 2022PHE135), and animal care was performed in accordance with the Guide for the Care and Use of Laboratory Animals set by the China Association of Laboratory/Animal Care.

### 2.2. Experimental design

In this experiment, 45 Wistar rats were randomly assigned to four groups: group 1 (*n* = 15) received a postauricular injection of NTG (10 mg/kg) (PI-NTG) every other day for 9 days; group 2 (*n* = 10) received a postauricular injection of 0.9% saline (10 mL/kg) (PI-NS) every other day for 9 days; group 3 (*n* = 10) received a intraperitoneal injection of NTG (10 mg/kg) (II-NTG) every other day for 9 days; and group 4 (*n* = 10) received a intraperitoneal injection of 0.9% saline (10 mL/kg) (II-NS) every other day for 9 days. The body weight was monitored every other day for all rats. Forepaw sensitivity to mechanical stimulation was assessed using von Frey device 20 min before and 2 h after each dose for all rats. Five rats were randomly selected from group 1 to continue feeding for 14 days after the last dose, all remaining rats underwent ABR test 2 h after the last dose. After 14 days, the five rats from group 1 were assessed again for forepaw sensitivity to mechanical stimulation and underwent ABR tests.

### 2.3. Drug administration

The formula of NTG for injection was prepared as described previously (9). 5.0 mg/ml NTG (Beijing Yiming, China) was diluted with 0.9% saline to 1 mg/ml. Rats in the postauricular group were injected in the middle of the right postauricular groove. Rats in the intraperitoneal group were injected into the lower left quadrant of their abdomen.

### 2.4. Behavioral observation

The behavioral activities of the rats were observed and recorded before and after each administration. NTG-treated animals showed more anxiety-like behavior such as increased self-grooming and face rubbing behavior (10).

### 2.5. Measurement of forepaw thresholds after mechanical stimulation

As a surrogate measure of pain/pronociception, tactile sensitivity to stimulation with von Frey monofilaments (North Coast Medical, USA) within fore paw nociceptive circuits was measured using the up-down paradigm (11) as previously described in detail (12). The testing was performed with the rat placed in clear plexiglas chambers on a mesh floor. Rats were placed in the chambers for acclimatization 30–45 min prior to testing. Calculation of 50% withdrawal thresholds was done using the free online calculator at https://bioapps.shinyapps.io/von_frey_app/ with application of inter-filament steps (13). Tactile sensitivity was measured at baseline and 120 min after each administration by a blinded experimenter.

### 2.6. Auditory brain stem response

ABR measurement was carried out in a sound-attenuating, electrically shielded booth located inside a sound-attenuating room. The rats were anesthetized with 10% chloral hydrate (4 mL/kg) injected intraperitoneal. ABR responses were recorded using subdermal needle electrodes. Needle electrodes were placed at the vertex (active), the test ear (reference), and the contralateral ear (ground) pinnae. Tucker Davis Technologies (TDT) System III hardware and SigGen/BioSig software (TDT, Alachua, FL USA) were used to present the stimulus and record responses. Click and 4, 8, 16, 24, and 32 kHz tone bursts were used as the auditory stimulant. Up to 1,024 responses were averaged for each stimulus level. Hearing thresholds were defined starting from 90 dB SPL, decreasing in 10 dB increments each time. Thresholds were interpolated between the lowest stimulus level where a response is observed, and 5 dB lower, where no response is observed. The latency time of I, II, III, IV, and V wave with 4 kHz 90 dB SPL tone bursts ABR waveform was recorded. Rats in the postauricular group tested both ears, while those in the intraperitoneal group only tested the right ear.

### 2.7. Statistical analysis

Descriptive data were presented as means and standard deviations (SD). Student's *t*-test was used for statistical comparisons between two groups. One-way or two-way ANOVA followed by *post-hoc* analysis with the Tukey test was used for statistical comparisons among groups. Mann Whitney *U*-test was selected for the non-parametric analysis. All statistical analyses were performed using SPSS software (version 27.0, IBM, USA), and statistical significance was set at *p* < 0.05.

## 3. Results

### 3.1. PI-NTG could induce migraine without affecting the animals' weight gain

**Figure 1:**
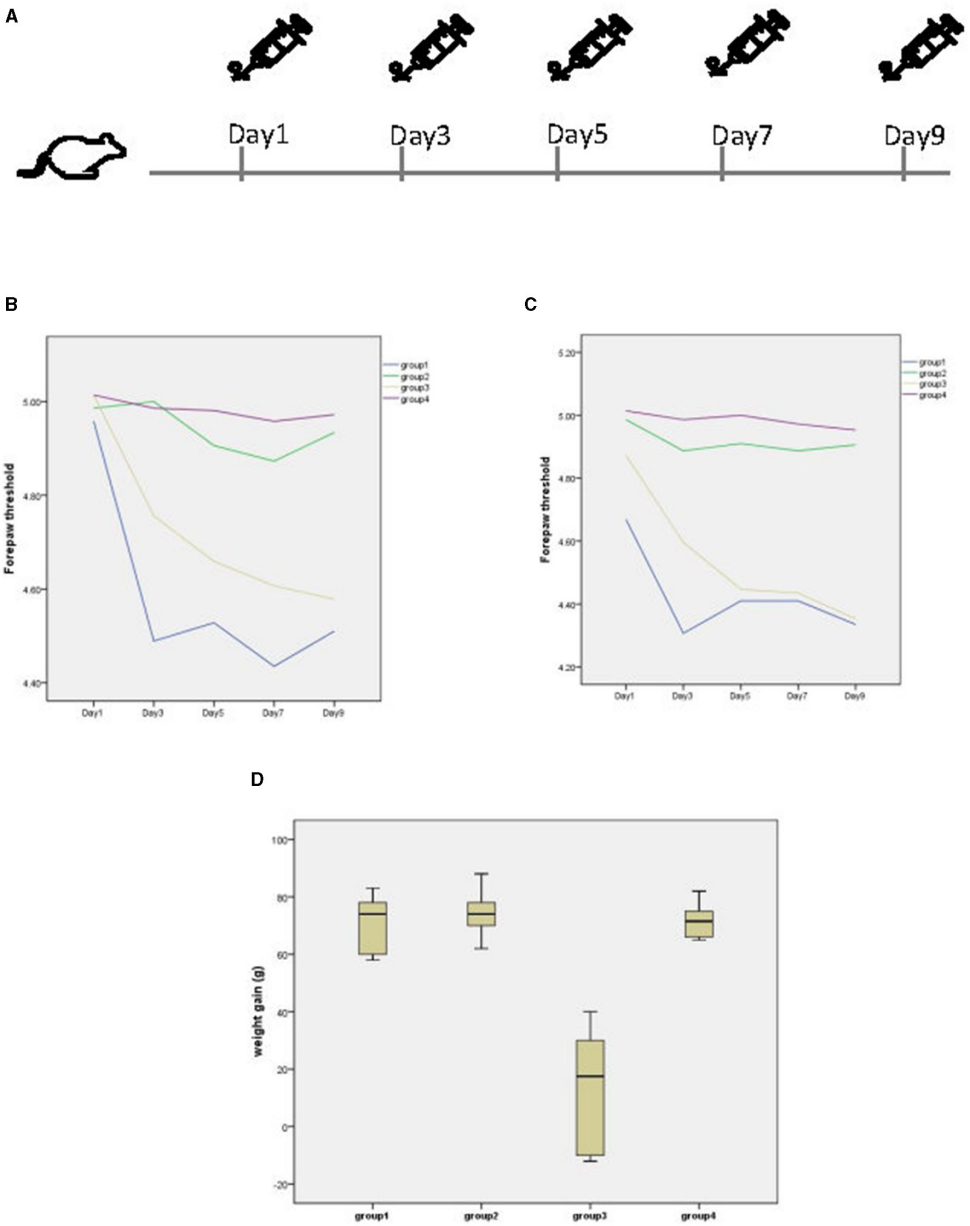
**(A)** Timeline of administration. **(B)** Basal and post-treatment tactile threshold **(C)** of forepaw were markedly decreased in a time dependent manner after NTG injection. **(D)** Body weight growth of each group, the weight gain is the weight on day 9 minus the weight on day 1. Group 1 (*n* = 10): postauricular NTG injection group; Group 2 (*n* = 10): postauricular saline injection group; Group 3 (*n* = 10): intraperitoneal NTG injection group; Group 4 (*n* = 10): intraperitoneal saline injection group.

The specific administration schedule is shown in Figure 1A. Behavioral manifestations related to migraine occurred after each administration of NTG. Chronic injection of NTG produced progressive basal hypersensitivity (Figure 1B) and acute allodynia (Figure 1C). However, abdominal pain, loss of appetite, and even diarrhea were observed in II-NTG rats, while no such phenomenon was observed in PI-NTG rats. During the modeling process, there was no significant difference in the body weight growth of rats in the PI-NTG group, the PI-NS group and the II-NS group (*p* > 0.05), while the body weight of rats in the II-NTG group increased slowly or even negatively, which was significantly different from other groups (Figure 1D; *p* < 0.05).

### 3.2. Compared with II-NTG group, PI-NTG rats showed more severe hearing loss

Compared with the II-NTG group, the ABR threshold of Click, 4 and 8 kHz in the PI-NTG injection group was increased (Table 1; *p* < 0.05), the latency was prolonged (Table 2; *p* < 0.05), and the ABR threshold in the right ear was higher than that in the left ear (Table 1; *p* < 0.05). There was no significant change in ABR threshold of rats in the II-NTG group, only the latency was prolonged.

**Table 1:** ABR threshold (dB SPL) $\left(\bar{x}\pm s\right)$.

	**Group1 R**	**Group1 L**	**Group2 R**	**Group2 L**	**Group3 R**	**Group4 R**
Click	39.50 ± 3.69	32.00 ± 2.58	30.00 ± 0.00	30.00 ± 0.00	23.00 ± 2.58	21.00 ± 2.11
4 k	33.50 ± 3.38	28.50 ± 5.30	25.00 ± 0.00	25.00 ± 0.00	18.00 ± 2.58	16.00 ± 2.11
8 k	22.00 ± 4.22	17.00 ± 4.22	17.00 ± 2.58	17.00 ± 2.58	10.00 ± 0.00	10.00 ± 0.00
16 k	10.00 ± 0.00	10.00 ± 0.00	10.00 ± 0.00	10.00 ± 0.00	10.00 ± 0.00	10.00 ± 0.00
24 k	10.00 ± 0.00	10.00 ± 0.00	10.00 ± 0.00	10.00 ± 0.00	10.00 ± 0.00	10.00 ± 0.00
32 k	10.00 ± 0.00	10.00 ± 0.00	10.00 ± 0.00	10.00 ± 0.00	10.00 ± 0.00	10.00 ± 0.00

Group 1 (n = 10): postauricular NTG injection group; Group 2 (n = 10): postauricular saline injection group; Group 3 (n = 10): intraperitoneal NTG injection group; Group 4 (n = 10): intraperitoneal saline injection group.

R, Right ear; L, Left ear.

**Table 2:** 4 kHz 90 dB SPL tone bursts ABR latency (ms) $\left(\bar{x}\pm s\right)$.

	**Group1 R**	**Group1 L**	**Group2 R**	**Group2 L**	**Group3 R**	**Group4 R**
I	1.38 ± 0.02	1.31 ± 0.06	1.31 ± 0.03	1.31 ± 0.04	1.33 ± 0.02	1.29 ± 0.05
II	2.26 ± 0.16	2.05 ± 0.16	2.15 ± 0.12	2.07 ± 0.07	2.13 ± 0.06	2.03 ± 0.11
III	3.00 ± 0.25	2.70 ± 0.12	2.77 ± 0.18	2.70 ± 0.08	2.79 ± 0.07	2.64 ± 0.10
IV	3.76 ± 0.27	3.56 ± 0.14	3.65 ± 0.33	3.57 ± 0.07	3.60 ± 0.11	3.48 ± 0.11
V	4.95 ± 0.42	4.49 ± 0.16	4.56 ± 0.06	4.53 ± 0.07	4.64 ± 0.16	4.46 ± 0.12

Group 1 (n = 10): postauricular NTG injection group; Group 2 (n = 10): postauricular saline injection group; Group 3 (n = 10): intraperitoneal NTG injection group; Group 4 (n = 10): intraperitoneal saline injection group.

R, Right ear; L, Left ear.

### 3.3. Hearing loss was irreversible in PI-NTG group

Five rats in the PI-NTG group were selected and fed for 14 days after the last administration. The tactile threshold of forepaw and ABR were measured again. The results showed that the paw mechanical pain threshold basically returned to the state before administration on day 1. The ABR threshold and latency did not change significantly from the day after the last dose.

## 4. Discussion

The mechanism of migraine accompanied with inner ear dysfunction remains unclear. However, there are few basic researches on the relationship between migraine and inner ear function, especially animal experiments on the effect of migraine on auditory function. Previous studies have found that the latency of ABR in migraine animal models changes, but the threshold does not change significantly (5). Therefore, it is necessary to establish better animal models with both migraine and hearing loss, so as to understand the relationship between migraine and inner ear function deeply.

The results of this study show that it is feasible to establish a rat model of migraine by postauricular injection of nitroglycerin. Every time after postauricular injection of nitroglycerin, the rats showed the corresponding behavioral manifestations and hyperalgesia of migraine. Chronic intermittent administration can cause progressive, persistent hyperalgesia. The results of this study showed that the rats in the intraperitoneal injection of nitroglycerin group had poor appetite, reduced food intake, slow weight gain, or even negative growth, which was significantly different from the postauricular injection of nitroglycerin group. Compared with the intraperitoneal injection of nitroglycerin, postauricular injection of nitroglycerin is safer and has fewer systemic side effects. However, some rats with postauricular injection of nitroglycerin showed hair loss on the postauricular skin.

The results of this study show that postauricular injection of nitroglycerin can increase the ABR threshold and prolong the latency of ABR in rats, while intraperitoneal injection of nitroglycerin has no significant change in the ABR threshold of rats, only show prolonged latency, suggesting that the migraine model rat induced by postauricular injection of nitroglycerin is more likely to cause inner ear auditory function damage. The results of this study showed that the increase of ABR threshold in migraine model rat induced by post-auricular injection of nitroglycerin mainly occurred in click and 4 kHz tone bursts. The increase of ABR threshold was also observed in 8 kHz tone bursts, but it was not as obvious as that in 4 kHz tone bursts. The ABR threshold of 16, 24, and 32 kHz tone bursts did not change. The results suggest that the hearing changes in the migraine model rat induced by post-auricular injection of nitroglycerin mainly occur in the low frequency hearing, and the high frequency hearing is not impaired, which is consistent with the hearing changes in most migraine patients clinically. Shi et al. (14) studied the hearing of 166 patients with vestibular migraine and found that the hearing impairment of VM patients was mainly manifested as low frequency hearing loss. Xue et al. (15) also found that patients with VM mainly presented with low-frequency hearing loss, and proposed that the history of migraine may be the cause of sudden low-frequency hearing loss.

The mechanism of migraine-related hearing loss is still unclear, and several theories have been proposed: (1) Migraine triggers vasospasm in the small arteries of the cochlea and labyrinth, which can induce endolymphatic hydrops (16). (2) Some inflammation and neurotransmitters involved in the pathogenesis of migraine affect the inner ear and central auditory system (17). (3) Ion channels expressed in both the inner ear and the brain may affect the peripheral and central auditory systems (18). The results of this study showed that the rat migraine model with postauricular injection of nitroglycerin was mainly characterized by low-frequency hearing loss, and there was no significant change in ABR hearing loss 14 days after administration. It was speculated that the rats in this model may have labyrinthiotic hydrocephaly. Due to the limitation of experimental time and conditions, this subject was not further verified by electrocochleogram. In addition, the possibility of ototoxicity should be considered, but intraperitoneal administration of nitroglycerin did not change the ABR threshold of rats, and the relevant literature did not mention that nitroglycerin had ototoxicity.

In 2018, with the cochlear migraine first proposed by Lai et al. (19), Cochlear migraine entered the field of view of the population. Cochlear migraine is a disease that is clinically related to migraine and mainly produces moderate to severe auditory symptoms. The results of this study show that the migraine model rat induced by postauricular injection of nitroglycerin accompanied with hearing loss, suggesting that this model is more inclined to cochlear migraine, and may be used as an animal model of cochlear migraine, it provides an important foundation for future clinical research.

## 5. Limitation

There were some limitations in our research. First, our study found that the migraine model rat induced by postauricular nitroglycerin injection was mainly characterized by low frequency hearing loss, and it was speculated that the inner ear of this migraine model rats might have hydrops of membranous labyrinth. However, due to the limitations of experimental time and conditions, this subject was not further verified by electrocochleogram, which is worthy of further study. Second, our study did not further explore the molecular mechanism of hearing loss in this model, which is well worth exploring. The last but not the least, we didn't have an effective mean of measuring vestibular function in rats to more fully evaluate inner ear function of this model.

## 6. Conclusion

In conclusion, we demonstrated that postauricular injection of nitroglycerin is a safer and more effective way to model migraine in rats than intraperitoneal injection. Postauricular injection of nitroglycerin has more damage to the auditory function of rats. Therefore, the migraine model rat induced by postauricular injection of nitroglycerin may be a new model of cochlear migraine. The animal model of migraine established by our new method not only validates the effect of migraine on hearing, but also lays a foundation for future clinical research.

## Data availability statement

The raw data supporting the conclusions of this article will be made available by the authors, without undue reservation.

## Ethics statement

The animal study was approved by Animal Ethics Committee of Peking University People's Hospital. The study was conducted in accordance with the local legislation and institutional requirements.

## Author contributions

RQ: Writing—original draft. JZ: Writing—review & editing. TD: Writing—review & editing. LY: Writing—review & editing.
